# Distributed Artificial Intelligence for Organizational and Behavioral Recognition of Bees and Ants †

**DOI:** 10.3390/s26020622

**Published:** 2026-01-16

**Authors:** Apolinar Velarde Martinez, Gilberto Gonzalez Rodriguez

**Affiliations:** 1 Departamento de Posgrado e Investigación, Instituto Tecnológico el Llano Aguascalientes, Aguascalientes 20330, Mexico; 2 Departamento de Ciencias Básicas, Instituto Tecnológico el Llano Aguascalientes, Aguascalientes 20330, Mexico; gilberto.gr@llano.tecnm.mx

**Keywords:** scene graph generation, graph neural network, behavioral recognition, internet of things, sensors, artrophods

## Abstract

Scientific studies have demonstrated how certain insect species can be used as bioindicators and reverse environmental degradation through their behavior and organization. Studying these species involves capturing and extracting hundreds of insects from a colony for subsequent study, analysis, and observation. This allows researchers to classify the individuals and also determine the organizational structure and behavioral patterns of the insects within colonies. The miniaturization of hardware devices for data and image acquisition, coupled with new Artificial Intelligence techniques such as Scene Graph Generation (SGG), has evolved from the detection and recognition of objects in an image to the understanding of relationships between objects and the ability to produce textual descriptions based on image content and environmental parameters. This research paper presents the design and functionality of a distributed computing architecture for image and video acquisition of bees and ants in their natural environment, in addition to a parallel computing architecture that hosts two datasets with images of real environments from which scene graphs are generated to recognize, classify, and analyze the behaviors of bees and ants while preserving and protecting these species. The experiments that were carried out are classified into two categories, namely the recognition and classification of objects in the image and the understanding of the relationships between objects and the generation of textual descriptions of the images. The results of the experiments, conducted in real-life environments, show recognition rates above 70%, classification rates above 80%, and comprehension and generation of textual descriptions with an assertive rate of 85%.

## 1. Introduction

Scientific research, through studies and observations, has demonstrated the existence of beneficial species that can reverse environmental degradation caused by industrial development and human overpopulation. Ants and bees are species that restore the environment [1] and serve as biological indicators of environmental change [2]. Biologists, entomologists, behavioral neurobiologists, and behavioral ecologists [3] have studied and observed behaviors such as conduct and organization in these species. Although these insects have intrigued specialists for many years due to their peculiar behavior and organizational structure, many questions remain unanswered, and studies on them continue with the goal of understanding the beneficial impacts they provide to the planet.

The benefits provided by these arthropods are numerous and varied. Ants are ecological and environmental indicators in ecological restoration and rehabilitation processes [4]. They constantly remove particles from the substrate where they live, favoring the flow of nutrients and their mineralization [5]; actively participate in seed dispersal [6,7]; offer better benefits in biological pest control as opposed to pesticides, which have no effect on some pest species that have developed resistance to pesticides [1]; and are used to predict the conditions of environmental conservation [8].

In the same way, honeybees are pollinators in intensive agricultural systems [9,10,11]. They produce foods with high nutritional value [12], such as honey and propolis, and they are also producers of pollen and wax [13]. Apis mellifera colonies are used as bioindicators as they allow environmental sampling of different nature [14]; other research works show that honey produced by bees can also be used as a bioindicator [13,15].

Studies targeting these insects involve trapping hundreds of insects from a colony, which are then subjected to containers of poisonous liquids, which cause harm or death when ingested, inhaled, or absorbed, causing their normal practices to be violated. To conduct ant studies in ant colonies, pitfall traps distributed in transects are used; pitfall traps consist of 100 mL plastic containers placed flush with the ground and partially filled with a 30% propylene glycol solution and a few drops of detergent to retain and preserve the intercepted ants; collected specimens are preserved in 96% ethanol [7,8]. In the same way, to conduct bee studies, two types of sampling are carried out: double-sieve sampling and powdered sugar sampling [16]. These samples imply opening of hives, and hundreds of bees are collected to be sacrificed with isopropyl alcohol and discarded with filters to verify the health status of the hive [17,18,19]. Another study [20] reports that bees are collected on the same day of the experiment, anesthetized, and briefly cooled in ice. The vast majority of studies are carried out in distributed sites in order to determine the diversity of species and different sites where the species live.

Each member of an ant or bee colony has specific roles to play. Some are workers, others are drones, and there is only one queen of the colony. The disappearance of individuals can jeopardize the organizational capacity of these arthropods and place the colony on the brink of collapse. For example, queens are insects that represent the entire population of the nest, so their mortality can put the entire colony on the brink of collapse. In certain ant species, smaller ants protect the food they find, while larger ones transport it back to the nest. Some are foraging workers, and others care for the young. Some specialize in collecting pollen, water, or tree resin, while others specialize in pheromone trails [21].

Technological advances in computer vision have gone from detecting and recognizing objects in an image, to understanding the relationships between objects and detecting a visual relationship between them, to generating a textual description and creating a caption of the image based on the image content [22]. A scene graphs is a structured representation of a scene that can express the objects, attributes, and social relationships between objects in the scene. Also, the miniaturization of devices with next-generation hardware operated with solar-powered electric devices can access remote sites to capture, store, process, and output data analysis results.

Considering the above and taking into account the advances in deep learning and the generation of scene graphs to recognize objects, attributes, and relationships between objects in the scene, it is possible to build a non-invasive artificial vision computational system to recognize, classify, and describe the organizational and behavioral behavior of insects while preserving and avoiding their mass sacrifice.

To observe organizational and behavioral characteristics, nest access is a little-explored location in research targeting ants, and in bee research, some work has focused on the beehive entrance [23,24]; some research records bee traffic and flower visits at the beehive entrance, providing interesting information on how several bees interact to take advantage of a common set of flowers. In this research, we consider nest access to be the best observation location because it is where all the inhabitants of that nest must go. They come out for the first time after birth, and the nest is the space through which food is brought closer and is the best-guarded location to prevent intruders from entering. Bee traffic at the nest entrance provides interesting information on how several bees interact to take advantage of a common set of flowers [25]. Thus, with a system installed at the nest entrance, it is possible to obtain information on distinctive life history traits and show distinctive responses to natural and anthropogenic disturbances.

To carry this out, two tools from the computational field are used: distributed computing and parallel computing. First, for distributed computing, we use miniaturized devices from the Internet of Things (IoT) with which images and videos of insects are obtained from remote locations; second, with high-performance parallel computing, we can design Distributed Artificial Intelligence that allows us to carry out automatic shape recognition and obtain knowledge of the behavior of living beings in uncontrolled environments. In addition to the above, in the extensive literature reviewed, a system that uses the miniaturization of computing devices and Artificial Intelligence techniques to conduct studies such as those proposed in this research, related to bees and ants, was not found. This paper is an extension version of the conference paper [26].

This research work is organized as follows: in Section 2, a set of works related to the study and observation of ants and bees, as well as applications of Scene Graph Generation, are described. The general objective and specific objectives of the project are listed in Section 3. The justification of the project is presented in Section 4. A set of definitions related to this work appears in Section 5; the formal definition of the problem is presented in Section 6; and the materials and methods are presented in Section 7. The Architecture of Distributed Artificial Intelligence is presented in Section 8. The experiments are described in Section 9. Finally, conclusions and discussion are presented in Section 10 and Section 11, respectively.

## 2. Related Works

In this section, a set of studies found in the literature related to scene graph generation are described. These studies have been used in this research project. The section is organized into three subsections: first, we present studies related to ants in different countries, which highlight the importance of ants as bioindicators and the parameters that drive ant behavior; second, we present studies related to bee behavior; and finally, we present studies related to scene graph generation.

### 2.1. Ants

The authors of [1] aim to understand the net effects of ants regarding biological control considering their services and disservices, hypothesizing that field size, crop system, and experiment duration would modulate the effects of ants on the abundance of pests and their natural enemies, plant damage, and crop yield; three variables were used, namely abundance of natural enemies, plant damage, and crop yield. The crops studied were different, with the most abundant being citrus crops (169 cases), mango (22 cases), apple, and cocoa (21 cases each). The characteristic observed in the ants was their size; they extracted information about the most abundant ant species in each study. They used the Global Ant Database [27] and additional literature to assess ant body length. They found that the size of the most abundant ant is a proxy for one important trait of this organism among many others.

The study presented in [8] aimed to evaluate the bioindicator potential of the ant fauna. This study aimed (a) to characterize the diversity of ants in different habitat strata in the Parque Estadual do Turvo located in Derrubadas, Rio Grande do Sul, Brazil, and (b) to analyze the bioindicator potential of ant species for soil and leaf litter and arboreal strata; the authors sampled the ant assemblage at five sites distributed along the road that crosses the park to the Uruguay River. Sampling methods were soil and canopy pitfall traps, sardine baits, glucose baits, entomological umbrellas, and a sweeping net. They recorded a richness of 157 species belonging to 32 genera and eight subfamilies. They observed that only nine species (5.7% of the sampled richness) had a significant indication value. The authors of the research consider that ant species can bioindicate soil cover, and the presence of leaf litter, vegetation diversity, and stage was confirmed.

A study aiming to provide a biological assessment using an ant-based multimetric index (MMI) sensitive to anthropogenic disturbances in riparian systems for monitoring, conservation, and restoration purposes, and to clarify the extent to which metrics based on ant responses provide useful information that cannot be provided by traditional physical and structural indicators, was carried out in [7]. The proposed study is divided into four steps: identify the river typology of the sampling sites, assess the pressure gradient for each river type, develop the ant-based MMI, and compare the results obtained using the new ant-based index with those of a traditional physical and structural index. The results presented 2268 individuals comprising 22 ant species and 13 genera, and four subfamilies were identified in the study area. With a collinearity analysis, six metrics showed significant differences between disturbed and less disturbed species in the upland river type, while three metrics allowed the separation between disturbed and less disturbed species in the lowland river type. For the upland river type, five main metrics were used: observed species richness, closed habitat species, large ants, cryptic ants, opportunistic ants, and T. caespitum foraging activity. For the lowland river type, foraging activity of ants, seed foragers, and A. senilis were assessed.

Considering that ants are easy to measure, easy to sample, sensitive to environmental stress, important in ecosystem functioning, and have a wide distribution, high abundance, and predictable responses to environmental stress, in [4], these insects were used as bioindicators to assess the ecosystem health of the Northern-Indian Shivalik mountain range through measurement of diversity, community patterns, species composition, and the influence of invasive species of Formicidae; studies were conducted in 75 sites from 44 locations in three habitats, namely primary forest (PF), secondary forest (SF), and non-forest areas (NF), using six collection techniques. The research obtained the most comprehensive dataset compiled for Indian ants to date (sample coverage 94% to 97%) and sampled 31,487 ant specimens, representing 181 species from 59 genera and 9 ant families.

In [28], research is conducted to explore benefits and limitations of applying a self-organization approach to ant social structures; it shows how self-organization captures some of the diversity of ant social patterns, and it presents the spatiotemporal structures in ant societies, considering an analogy with self-organization patterns observed in physical and biochemical systems. The authors choose the myrmecological pattern of foraging to examine several properties that are typical features of self-organization. The study shows how fluctuations, colony size, and environmental parameters influence the dynamics of feedback loops between interacting ants and thus shape the collective response of the entire ant society. It highlights the capacity of each ant to process information and how ants adjust their interactions with their nestmates, as well as the functional properties of social patterns. The results obtained show that self-organization is a powerful set of pattern-generating mechanisms, or, in other words, a potent generator of biological diversity. Self-organization provides an answer to a challenge in evolutionary biology: how can we generate a wide variety of social patterns in group-living animals while limiting the number of behavioral rules and physiological attributes that should be “encoded” in individuals.

Reference [29] uses ecologically relevant behavioral traits to assess colony-level behavioral syndrome in rock ants. Using field and laboratory assays, they measure foraging effort, colony responses to different types of resources, activity level, threat response, and aggression. In their research, the authors find a colony-level syndrome that suggests that colonies differ consistently in their coping style: some are more risk-prone, while others are more risk-averse. Also, by collecting data across their study areas for this species in North America, they find that environmental variation can affect how different populations maintain consistent variation in colony behavior.

An extensive study of ant–plant interactions is presented in [30]; this work investigates ant behavior controlled by plant volatile organic compounds (VOCs). Through in situ experiments, it is shown that pollen-derived volatiles can specifically and transiently deter ants during dehiscence; this study proposes the existence of three main types of adaptations that many plant species use to protect their valuable floral structures from ants: architectural barriers; decoys and bribes (e.g., food or lodging located some distance from the flowers); and chemical deterrents, often using floral volatile organic compounds (VOCs).

In [21], the various communication methods used by ants to direct their nest mates to feeding sites, as well as their recruitment behavior for other foraging ants through dances or direct physical contact at the nest, are studied. A broad analysis of the quantities and types of pheromones that ants emit while searching for food is described, in addition to the communication behaviors and interactions that ants perform through pheromone secretion. The functions that each individual in the colony performs are broadly established in this research.

Other research works have carried out studies of ant behavior using mathematical tools, for example, in [31], a process algebra approach to modeling ant colony behavior is described; the objective of the work is to show how process algebras can be usefully applied to understand the social biology of insects and to study the relationship between the algorithmic behavior of each insect and the dynamic behavior of its colony.

### 2.2. Bees

A wide range of studies on honeybee behavior and its use as bioindicators can be found in the literature. In this subsection, we summarize some of the work related to these two topics. The focus of each research project and the results obtained in each area are described.

A comprehensive set of experimental protocols for detailed studies of all aspects of honey bee behavior is presented in [32]. In this research, honey bees are prepared for behavioral assays to quantify sensory response to gustatory, visual, and olfactory stimuli, appetitive and aversive learning under controlled laboratory conditions, and free-flight learning paradigms; this is carried out to investigate a wide range of cognitive abilities in honey bees. To explore changes in temperature, experiments analyzing honey bee locomotion across temperature gradients are presented and described in this study.

A study aiming to carry out precision beekeeping that analyzes the development of integrated monitoring systems with multiple sensors and automated motion tracking devices to connect information on bee behavior, hive functioning, and the external environment, in order to explore new fields in bee biology, is presented in [25]. Within this study, the parameters and technologies for obtaining long-term data on the behavior and ecology of bees inside and outside the hive are listed. Through these systems, the behaviors studied include in-nest behavior and foraging activity, foraging activity and spatial behavior, and spatial behavior and social interactions.

Other works address behavioral aspects of bees, such as [33], where the evolutionary history of eusociality and the antiquity of eusocial behavior in apid bees are studied, as well as swarming behavior (characteristics of highly eusocial colonies) and the organization and genetic basis of social behavior in honey bees and their relatives. In [20], an animal model was developed to study how the immune and nervous systems interact in a coordinated manner during an infection in honey bees; the study was carried out by administering a bacterial lipopolysaccharide (LPS) into the thorax of honey bees. This research arises from the assumption that animals undergo various changes in their normal physiology and behavior, which may include lethargy, loss of appetite, and reduced grooming and general movement during the presence of a disease.

A system for automatically analyzing and labeling honeybee movements by creating a behavioral model from examples provided by an expert is presented in [3]. Starting from a manually labeled training dataset, the system automatically completes the entire behavioral modeling and labeling process. Experiments involve video recording activity in a hive with a vision-based tracker; this information is considered the location of each animal, and then numerical features such as speed and direction change are extracted. By combining kernel regression classification techniques and hidden Markov models (HMMs), the behaviors generated by the movements are recognized, the sequence of movements of each observed animal is labeled, and evaluations are performed with hundreds of honeybee trajectories.

Considering honeybees as eusocial insects, reference [34] studies how gene regulatory network (GRN) activity influences developmental plasticity and behavioral performance at the individual level among worker bees. The proposed study tests the hypothesis that individual differences in behavior are associated with changes in brain GRN activity (i.e., changes in the expression of transcription factors and their target genes). Making use of automatic behavior tracking, genomics, and the broad behavioral plasticity present in honeybee colonies with laying workers, this research presents results with key insights into the mechanisms underlying the regulation of individual differences in behavior by brain GRNs.

### 2.3. Studies Related to Scene Graph Generation

This section provides a summary of some articles related to scene graph generation, which have served as the basis for this research work.

We start from two comprehensive surveys of scene graphs (consulted in [22,35]), which contain extensive references and analyses of published research papers. Current methods for generating images based on scene graphs are presented in [36]. Evaluation metrics, examples of deep learning benchmark datasets, and knowledge graph-based approaches are presented in [37]. In [38], a set of SGG applications is listed, such as image retrieval, visual reasoning, visual question answering, image captioning, structured image generation, and robotics. We have found GGS models in [39], where a relational embedding module is proposed that enables a model to jointly represent connections between all related objects rather than focusing on a single object, which benefits the classification of relationships between objects. In [40] a framework for semantic image retrieval is developed with two abstractions: a scene graph to describe the scene, and a scene graph base that associates the scene graph with the image.

Finally, in [41], less-explored areas are mentioned, such as the detection of distant object relationships and the detection of social relationships between humans to infer social ties, in addition to extracting hidden social interactions from visual data.

## 3. Project Objectives

### 3.1. General Objective of the Project

The objective of this study is to design a distributed computing architecture with IoT devices and a parallel computing architecture to acquire and process images and videos of insects in their natural environment to recognize, classify, and analyze their living behavior at nest entrances for the sake of preserving and protecting species that are beneficial to humans and the environment.

### 3.2. Specific Objectives

1. Construct the visual dataset “Bees”, consisting of 100 images with 15 objects, 12 attributes, and 15 relationships between pairs of objects to allow the modeling of the behavioral relationships of bees at the nest entrance.
2. Construct the visual dataset “Ants”, consisting of 100 images with 15 objects, 12 attributes, and 15 relationships between pairs of objects to allow modeling of the behavioral relationships of ants at the nest entrance.
3. Build three computational prototypes to observe groups of insects in their nests in three different geographical positions (sites) and determine whether three climatic conditions (temperature, humidity, and wind speed) are determining factors in the behavior of the observed insects.
4. Develop image descriptions, object descriptions, object attributes, relationships between objects, and question-and-answer pairs.
5. Recognize the complex shapes of insects to identify the type of living being observed through automatic recognition of artificial vision shapes.
6. Observe insect behavior at the nest entrance to recognize and classify distinctive life history traits (e.g., behavioral dominance, main food resources, and daily activity rhythm) and consequently show distinctive responses (e.g., abundance and species richness) to natural and anthropogenic disturbances.
7. Use automated computing resources to observe and analyze insects and prevent the mass killing of insects while helping to preserve and care for species that are beneficial to humans and the environment.

## 4. Justification of the Research Project

Observing and recognizing the life forms, behavior, and organizational structures of insects such as bees and ants is possible through the use of mechanical, electrical, and electronic devices operated by computer software. This is carried out to prevent collateral damage to the population, the elimination of individuals representing the colony, and killing of insects. Therefore, developing a computer system that automatically recognizes and classifies objects in an image helps provide an understanding of the relationships between objects and produces a textual description based on the image content; thus, it is justified for the sake of species preservation.

## 5. Preliminaries

This section presents a set of definitions, which are used in the following sections: Section 5.1 presents the terminology related to scene graphs, and in Section 5.2, the terminology used for scene graph construction is presented.

### 5.1. Scene Graph Terminology

Formally, a graph *G* contains a set of nodes *V* and a a set of edges *E*; each edge $e_{i\to j}\in C_{rel}$ defines a relation predicate between the subject node $V_{i}$ and the object node $V_{j}$. $C_{obj}$ is a set of object classes, and $C_{rel}$ is a set of relations [39].

The scene graph (SG) is a direct graph data structure and is defined as

(1) SG=(O,R,E)

where $O=\{o_{1},\ldots ,o_{n}\}$ represents the set of objects detected in the image and *n* is the number of objects. Every object can be denoted as $o_{i}=\left(c_{i},A_{i}\right)$, $c_{i}$ represent the category of the object, and $A_{i}$ are the attributes of the object.

*R* are relationships between nodes, i.e., relationships between the *i*-th and *j*-th object instances are expressed as $r_{i\to j},i,j\in \left\{1,2,\ldots ,n\right\}\;.$

*E* represents the edges between the object instance nodes and relationship nodes, so there are at most n×n edges in the initial graph; then, E⊆O×R×O.

Considering the representations of scene graphs, *I* represents a given image, and the SGG produces as output a scene graph with the instances of the objects found in the image, represented with the bounding boxes and the relationships that may exist between the objects. According to [22], we represent the above as

(2) $$SG_{O,R,E}^{I}=SGG\left(I\right)$$

### 5.2. Scene Graph Construction

**Figure 1:**
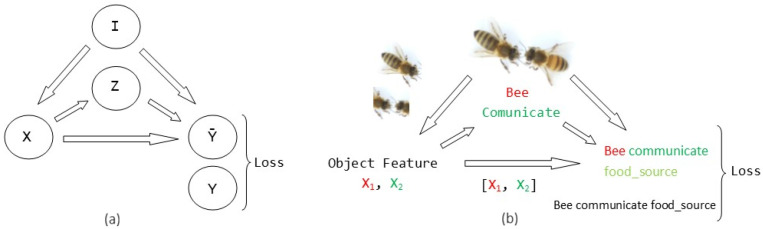
Process of building a scene graph and its corresponding application.

To define the process of building a scene graph, consider Figure 1. Figure 1a is a graph with five nodes and five edges (as the example of [22]), where *I* represents an input image, *X* represents the feature of the object and node *Z* represents the category of the object. and *Y* represents the category of the prediction predicate and the corresponding triple 〈subject−predicate−object〉. A fusion function acts as a receiver of the outputs of the three branches and generates the final quantile function, associated with the standard logistic distribution. Figure 1b exemplifies the construction of a scene graph for a behavior exhibited by bees at the entrance of the hive, using the graph from Figure 1a of this same figure.

Object Feature Extraction represented by the link I→X. Using the technique proposed in [22], a set of bounding boxes are extracted with their corresponding features; then, $B=b_{i}|i=1,\ldots ,m$, represents the set of bounding boxes, and $X=x_{i}|i=1,\ldots ,m$ represents the corresponding features of the input image *I*. The process of obtaining the characteristics is represented as

(3) $$Input:\left\{I\right\}\Rightarrow Output:\left\{x_{i}|i=1,\ldots ,m\right\}$$

This process yields the encoded visual context of each object in the image. In Figure 1b, two bounding boxes are extracted and presented in the two left-hand images: the isolated bee and the two bee heads.

Object Classification represented by the link X→Z. This process is expressed in the form

(4) $$Input:\left\{x_{i}\right\}\Rightarrow Output:\left\{z_{i},z_{i}\in O\right\},i=1,\ldots ,m$$

As an example case, we classify the image as a “Bee Communicate” in the image (greeting between bees) after object feature extraction is constructed; Section 7.2 shows this example in more detail.

Object Class Input for SGG represented by the link $Z\to \bar{Y}$. Here, the paired object label is used $\left(\bar{z_{i}},z_{j}\right)$, and a predicate $\bar{y_{ij}}$ between a pair of objects is predicted by a level *M*, called the combined embedding layer. The process can be expressed as

(5) $$Input:\left\{\left(z_{i},z_{j}\right)\right\}⟹Output:\left\{{\bar{y}}_{ij}\right\},i\neq j;i,j=1,\ldots ,m.$$

A priori knowledge and a priori statistics are computed in this step [22]. So, for our example, with the a priori knowledge of the image and the knowledge established in the region description (defined in the Section 7.2), a query is performed to retrieve images depicting scenes similar to the one described in the graph. The agreement between the retrieved images and the unannotated image (scenario) [40] is measured; to determine the agreement, a conditional random field is constructed that models the distribution of all possible relations, an inference set as the maximum posterior finds the most likely agreement, and the probability of the maximum a posteriori inference is taken as the score that measures the agreement between the retrieved images and the unannotated image.

Object Feature Input for SGG represented by the link $X\to \bar{Y}$. The object features, represented by means of paired $\left[x_{i},x_{j}\right]$ allows for the prediction of the corresponding predicate. The process is represented as

(6) $$Input:\left\{\left[x_{i},x_{j}\right]\right\}\Rightarrow Output:\left\{{\bar{y}}_{ij}\right\},i\neq j;i,j=1,\ldots ,m.$$

This link uncovers the entire context of the information. For the proposed example, we consider all the information obtained by applying all the relationships obtained with the conditional random field as the object feature input for SGG.

Visual Context Input for SGG represented by the link $I\to \bar{Y}$. The visual context feature $v_{ij}=Convs\left(RoIAlign\left(I,b_{i}\right)\cup b_{j}\right))$ [22] of the joint region $b_{i}\cup b_{j}$ is extracted in this link and predicts the corresponding triplet. This process is represented as

(7) $$Input:\left\{\left[x_{ij}\right]\right\}\Rightarrow Output:\left\{{\bar{y}}_{ij}\right\},i\neq j;i,j=1,\ldots ,m.$$

In the example in Figure 1b, the corresponding triplet is food_source, which constructs the predicate as “Bee Communicate food_source”.

**Training Loss:** Most of the proposed SGG models are trained using conventional cross-entropy losses for the object label and the predicate label; this training method is used in this work. To prevent a single link from spontaneously dominating the generation of logits $\bar{Y}$ [22], auxiliary cross-entropy losses are added to predict individually $\bar{Y}$ from each branch.

## 6. Formal Definition of the Research Problem

Considering the above definitions, formal definition of the research problem is defined as follows.

Given an image *I* containing numbered objects of the form $o_{1},o_{2},\ldots ,o_{n}$ which belong to the set θ of known objects and are represented by numbered graphs *G* of the form $G_{1},G_{2},\ldots ,G_{n}$, the goal is to find *n* hidden relationships between the objects of the form:

$$\begin{array}{cccccc} o_{1} & \overset{R}{\to } & o_{2}, & o_{3}, & \ldots & o_{n}\\ o_{2} & \overset{R}{\to } & o_{1}, & o_{3}, & \ldots & o_{n}\\ . & . & . & . & . & .\\ . & . & . & . & . & .\\ . & . & . & . & . & .\\ o_{n} & \overset{R}{\to } & o_{1}, & o_{3}, & \ldots & o_{n-1} \end{array}$$

The goal is to textually describe the image *I* using formal language. Then, the following objectives are met:

- Statement 1 $\left(S_{1}\right)$, object recognition. Given *I*, obtain $o_{1},o_{2},\ldots ,o_{n}$, where $o_{1},o_{2},\ldots ,o_{n}\in \theta$; otherwise, any $o_{n}\notin \theta$.
- Statement 2 $\left(S_{2}\right)$, object relationships. If $S_{1}$ is true, then for each $o_{1},o_{2},\ldots ,o_{n}$ create a graph *G* numbered $G_{1},G_{2},\ldots ,G_{n}$ and create the bounding boxes contained in the image. Find a relationship $R_{1},R_{2},\ldots ,R_{n}$ between
  $$\begin{array}{cccccccc} o_{1} & \overset{R}{\to } & o_{2}, & or & o_{3}, & or & \ldots & o_{n}\\ o_{2} & \overset{R}{\to } & o_{1}, & or & o_{3}, & or & \ldots & o_{n}\\ . & . & . & . & . & . & . & .\\ . & . & . & . & . & . & . & .\\ . & . & . & . & . & . & . & .\\ o_{n} & \overset{R}{\to } & o_{1}, & or & o_{3}, & or & \ldots & o_{n-1} \end{array}$$
- Statement 3 $\left(S_{3}\right)$, textual description. If and only if $S_{1}$ and $S_{2}$ are true, then the process of building a scene graph is realized and a formal description of *I* is generated.

## 7. Materials and Methods

In this section, all the electrical, electronic, and mechanical materials used in the experiments are described. The methodological approach for constructing the bee image dataset and the ant dataset is then described in detail. Likewise, the way in which a scene graph is produced and applied is described.

### 7.1. Materials

The materials used in this research are classified as materials related to the IoT architecture of distributed computing, materials for generating solar energy at the insect observation sites, the hardware for the parallel computing architecture, and the hardware for the aerial device used. Each of these materials is explained in the following sections.

The distributed computing IoT architecture, called observation prototypes, consists of four sets of devices used to acquire images from the arthropod nests. Each set of devices contains the following elements:

1. Three Raspberry Pi cards, each with 16 GB of memory and an Intel processor with WiFi connectivity. Technical information: Raspberry Pi 4 Computer Model B Version 8 GB RAM (Sony UK Technology Centre, Wales, UK).
2. Three 4.5 Mega Pixel Raspberry Pi high-definition cameras. Technical information: Raspberry Pi High-Quality Camera 12.3 MP7.9 mm diagonal image size. Raspberry Pi Foundation (UK charity/company) is Cambridge, UK.
3. Three Arduino UNOs, connected to a Raspberry Pi board, which contains two connected sensors, one for humidity and one for temperature. Technical information: Smart Projects, Ivrea, Italy, based on the ATmega328P.
4. Three Protoboard Breadboard 400 points for Arduino. Technical information: Total Tie Points: 400 (300 in the terminal strip area and 100 in the power distribution strips). Dimensions: Approximately 8.2–8.5 cm × 5.4–5.5 cm. Shenzhen iSmart Electronic Co., Ltd. (branded as ISMARTELEC) Chinese manufacturer (Shenzhen, China).
5. Three DHT11 temperature and humidity sensors. Technical information: Power Supply: 3.3V to 5.5V DC, Operating Voltage/I/O: 3V to 5, Temperature Range: 0 °C to 50 °C, Humidity Range: 20% to 80% RH, Humidity Accuracy: ±5% RH, Sampling Rate: 1 Hz (1 reading per second). Guangzhou, China.

**Figure 2:**
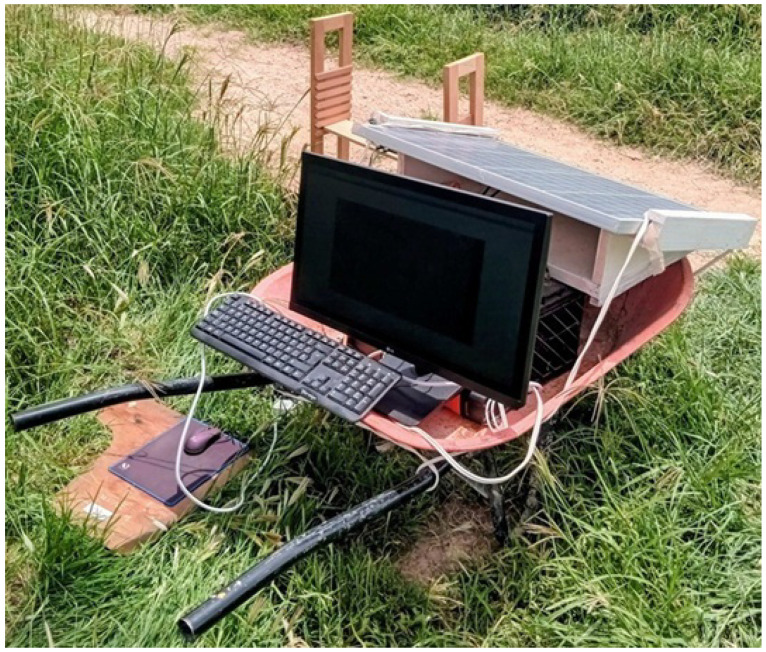
Observation prototype used to obtain real-time comparative data on the behavior of insects in three different sites.

From each of the four sets of devices, a device is extracted and an observation module is built consisting of a Raspberry Pi card, a high-definition camera, an Arduino UNO, a breadboard, and a temperature and humidity sensor; the image in Figure 2 shows an observation prototype already built. Each prototype is installed in one of the observation sites, which operate at the same time to obtain real-time comparative data on the behavior of insects in three different sites, where the nests are located. By installing the three observation modules, we can obtain visual information and data in a distributed and parallel manner.

To generate the energy needed to power each of the observation modules, the following materials are required:

1. Three solar panels, with the following technical information: Smart Projects, Ivrea, Italy 50 W, 12 Vdc, Polycrystalline, and 36 Grade A Cells connected to a voltage regulator and an inverter.
2. An Snc 20 Solar Controller 12/24v Display Kit Plant Lth Cale.
3. A 750W power inverter with USB ports and a DC and AC inverter (product brand: Truper).
4. A gray pneumatic tubular wheelbarrow, 5.5ft3, Pretul 20646, which is the mobile container used to transport the solar energy generator.

The mobile container is transported to the observation sites by the researcher using brute force.

With regard to parallel computing architecture, it consists of the following devices installed in the data processing center (CPD):

1. A DELL EMC Intel(R) Xeon(R) Silver 4210R CPU 2.40 GHz 20 Cores server, with the host operating system LINUX Ubuntu Server 24.04 LTS.
2. A Server Power Edge R330 Intel(R) Xeon(R) CPU E3-1220 V5 3.00 GHz, with the host operating system LINUX Ubuntu Server 24.04 LTS.
3. A Switch Cisco Gigabit 10/100/1000 for server interconnectivity.
4. An uninterruptible power supply, which supplies power to the servers and guarantees power supply in the event of a power failure.

In addition to the above, a DJI MINI 4 PRO aerial device (drone) is used to fly over the study area. This device is used to acquire the positions of the anthills to be observed. The aircraft flies within 180 hectares of university land.

### 7.2. Method

Using the proposed hardware, a methodological approach was developed, consisting of five steps. The steps are listed below, and each is explained in the following paragraphs:

1. Establishing the study area to carry out experiments with honeybees and ants.
2. Creating the dataset.
3. Generating a scene graph.
4. Training the neural network.

#### 7.2.1. Establishing the Study Area to Carry out Experiments with Honeybees and Ants

To collect visual information on the bees, there are two test apiaries at the university, which were used in this research project. To acquire information from other apiaries, the image acquisition equipment was brought to the site.

To locate the anthill locations and establish access points to the nests, a drone equipped with a video camera and transmitted to a cell phone was used. The videos acquired during the overflights were analyzed to detect the anthills’ geographical locations. Once the anthills were located, the image acquisition team moved to the positions found to collect the visual information.

Table 1 shows images of four examples of geographical locations and aerial photos of the four sampling sites at the university. The coordinates of the university where we conducted the research are 21.818966302777547,−102.10079656103468. The red circles show the exact locations of the anthills in the images. The sampled sites correspond to different lands; for example, image 1 corresponds to an anthill between a corn crop area and a bean crop area. Image 2 shows an anthill near a tree and next to pig and sheep farms. Image 3 is an anthill located in an area with cactus plants, where ants do not have access to the crops. Image 4 shows an anthill near a building and walkways for people.

#### 7.2.2. Creating the Dataset

There are methods proposed in the literature for the creation of datasets [22,42]. In this work, the following factors for the generation of the datasets and SGG were considered: images acquired at the observation sites; the descriptions of regions, objects, attributes, and relationships; the generation of region graphs; the generation of scene graphs; and the question–answer pairs. The way in which each of these components is applied to the construction of the “Bee” and “Ants” datasets is described in the following paragraphs.

Images acquired at the observation sites: Table 2 contains examples of images obtained from access to hives in different apiaries, and Table 3 shows images of entrances to different anthills. To more easily identify each image in the tables in the following explanations, each image from left to right, starting from the top left corner, is assigned a number; in addition to the number, each image is assigned a name related to the behavior that the bees show. Therefore, Table 2, Image 1, labeled “Greeting”, shows two bees greeting each other; Image 2, “Bee Type”, shows two types of bees, the Italian honey bee and the Africanized honey bee, distinguished by their yellow and black abdomen; Image 3, “Carrying Pollen”, shows a bee carrying pollen on its limbs; Image 4, “Intruder”, shows an intruder (a fly) at the hive entrance; Image 5, “Big Population”, shows a subset of bees on the hive access platform; and Image 6, “Grouping”, shows a group of four bees transmitting information about potential food sources.

The ants in Table 3 have been assigned a number from left to right, starting from the top left corner in the same way; the name assigned to each of the images corresponds to the behavior that the ants show.

Region Descriptions: Explaining or generating the description of an image is called image captioning or frame captioning [37]. The descriptions of the scene regions were made by experts and located by bounding boxes; overlaps between regions are allowed when the descriptions differ, with each sentence describing each of the regions varying from 1 to 16 words in length. Five descriptions of regions per image were considered. Table 4 illustrates the descriptions of two images that appear in Table 2: Image 1, “Greeting”, and Image 4, “Intruder”. For the sake of simplicity, in the rest of this document, we use the word “access” to refer to the beehive entrance or anthill entrance.

The descriptions of the scene regions, in the case of the ants, are considered image 1, “Greeting”, and image 5, “Big Population”. Table 5 illustrates the descriptions of two images that appear in Table 3. Because ants have more limited interactions at the nest entrance, in this research, only 5 descriptions of the regions of two scenes are considered.

Multiple objects and their bounding boxes: Once the bounding boxes have been drawn on the images, an average of 5 objects are detected within them; each object is bounded by a tight bounding box, and each object is canonicalized to a synset identifier in WordNet [43]. For example, bees would be mapped to bee.n.03 (which is the generic use of bee to refer to a bee), and the mellifera bee would be mapped to bee.n.01 (a bee). These two concepts can be joined to bee.n.01 since it is a hypernym of bee.n.03. Standardization is achieved with the WordNet ontology for naming an object with multiple names (e.g., bee, apis mellifera, and apis mellifera bee) and for connecting information between images. Table 6 shows the mapping from sentences to bounding boxes for the two images in Table 1; from item (a) to item (e), each sentence is mapped to describe each region of the bounding boxes.

The same is true for ants, where the bounding boxes are drawn on the images, with an average of three objects; each object is bounded by a tight bounding box, and each object is canonicalized to a synset identifier. For example, ant would be mapped to ant.n.03 (which is the generic use of ant to refer to an ant), and emmet bee would be mapped to ant.n.01 (an ant), since it is a synonym of ant.n.03. Just like the terms used to refer to a bee, we refer to an ant by standardizing with the WordNet ontology for naming an object with multiple names to connect information between images. Table 7 shows the mapping from sentences to bounding boxes for the two images in Table 3; from item (a) to item (e), each sentence is mapped to describe each region of the bounding boxes.

A set of attributes: Once the objects are collected from the region descriptions, the attributes associated with these objects are also collected. The attributes are canonized in WordNet [43]. Table 8 shows three attributes, color, state, and position, associated with the objects: Bee, Intruder, and Beehive Access.

In the case of ants, the selected attributes have been matched to the types of ants observed; in our case, the colors of the ants are red, yellow, and black. However, it is very difficult for intruders to approach the access point in an anthill because ants display very aggressive behavior and are very jealous of their spaces. Within the dataset, we have recorded the arrival of intruders to the access points of the anthills. In the case of access to the anthill, the attributes are similar to those of bees; access can be Free, Occupied, or Tumult, with their respective associated attributes. Table 9 shows objects and attributes associated with objects (ants).

**Figure 3:**
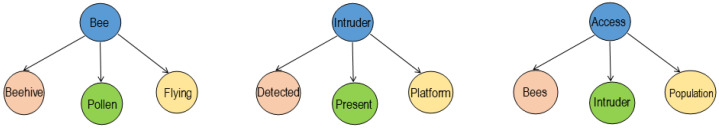
Examples of relationships which connect subject to object.

Set of relationships: Relationships connect objects with descriptive verbs, propositions, actions, comparisons, or propositional phrases. A relationship is directed from one object to another, that is, from the subject to the object. Relationships are canonicalized to an identifier from a WordNet synonym set [43]. Figure 3 shows three examples of relationships that connect the subject to the object. In example 1, the object Bee is related to three objects: Beehive, Pollen, and Flying.

**Figure 4:**
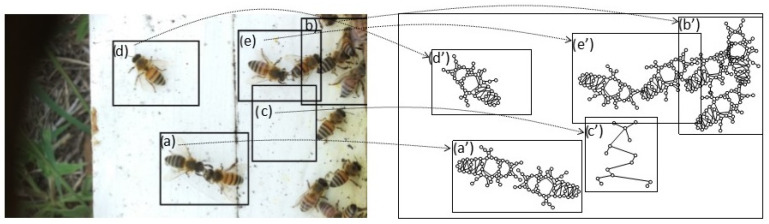
Example of a set of region graphs produced for Image 1 in Table 2. Each region is mapped to a region graph. In this image, for each of the regions (a–e) a region graph (a’–e’) is produced.

Set of region graphs: In this stage, a graph representation that addresses each of the regions is created; a region graph represents a structured part of the image. Objects, attributes, and relationships are represented by the nodes of the graphs. Objects are linked to their attributes, and relationships link one object to another. Figure 4 shows an example of the set of region graphs produced for Image 1, namely, Greeting from Table 2.

**Figure 5:**
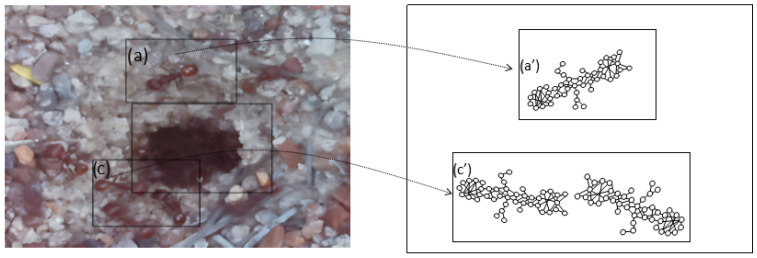
Example of a set of region graphs produced for Image 1 in Table 3. Each region of the image is assigned a region graph. In this image, for the two regions (a) and (c) a region graph is produced and labeled with (a’) and (c’).

The set of region graphs of ants is also shown in the Figure 5. The way in which the isolated graphs are created at the nest entrance and the ant greeting, which are shown in Table 3, are also exemplified in this figure.

#### 7.2.3. Scene Graph Generation

Scene Graph: Of the existing methods for scene graph generation in the literature [22,35], in this work, we used the Graph Neural Network (GNN)-based method [44,45,46] for the following justifications: First, in [19,26], a procedure for converting the entire image to a graph is described; by using the described method, in this research, a set of graphs are constructed in the step of generating the set of region graphs (described in the previous paragraphs), where each region is considered a graph. Then, using the above method, the region graphs are combined into a single scene graph that represents the entire image, that is, a scene graph is the union of all the region graphs and contains all the objects, attributes, and relations of the description of each region, which allows for multiple levels of scene information to be combined in a more coherent way. Section 7.2.4 describes how the neural network is trained, including the input to the neural network, the embedding vectors, and the generation of the network’s outputs.

**Figure 6:**
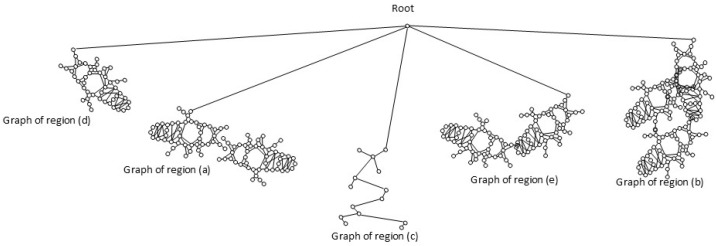
Example of a set of region graphs produced for Image 1 in Table 2. In this example, the set of region graphs, labeled (a–e) are combined to produce a single scene graph labeled as Root.

Figure 6 is an example of the set of region graphs produced for Image 1 in Table 2, combined into a single scene graph. Once the scene graph has been generated, graph reasoning is performed to complete the procedure; in this paper, the complete procedure for graph reasoning is described in following paragraphs.

**Figure 7:**
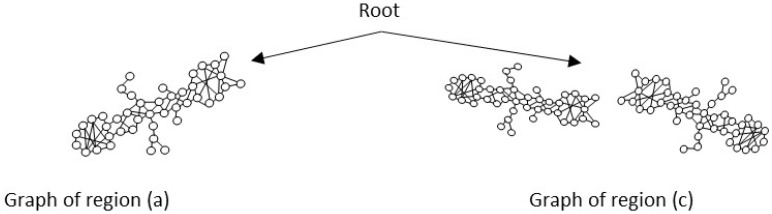
Example of a set of region graphs produced for Image 1 in Table 3. In this example, the region graphs, labeled (a,c) are combined to produce a single scene graph labeled as Root.

For the case of the ant scene graph, Figure 7 shows the set of region graphs produced for Image 1 in Table 3.

Set of question-and-answer pairs: Once the procedure for generating scene graphs from the images is complete, the final component is defining the set of question–answer pairs. To define the set of question–answer pairs, two types of questions and answers are associated with each image in the dataset; the first pair is free-form and based on the entire image; the other pair refers to questions and answers based on selected regions of the image. The question types per image are what, where, how, when, who, and why.

Table 10 shows a set of free-form questions and answers based on the complete image based on the full image. The results obtained with this example are described in Section 9, Experiments.

Graph Reasoning

This section describes graph reasoning for sentence detection, predicate classification, and scene graph classification, as referenced in [41].

Phrase detection: In this phase, an output text known as a label is generated 〈subject−predicate−object〉. The process for detecting relationships between the subject and the object is located using a bounding box, as defined in Section 5, Preliminaries, and is referenced in Table 3.

Classification of predicates: For this phase, a set of object pairs is generated within the image, multiple objects, and their bounding boxes, as explained in Section 7.2.2; from this set of object detected pairs, it is determined whether the pairs interact and the predicate of each pair is categorized.

Classification of scene graphs: Scene graphs take as input the located objects, predict the categories of the objects, and predict the predicates in each pairwise relationship.

The components of the method have been described so far; the following subsections explain the training of the neural network from scene graph generation and the evaluation method.

#### 7.2.4. Training the Neural Network

**Figure 8:**
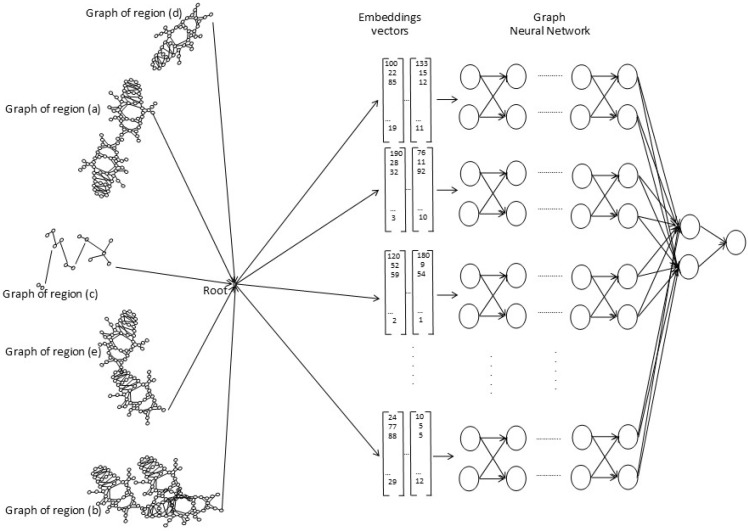
General scheme of the neural network training, consisting of the graph regions labeled from (a) to (e), the scene graph labeled as Root, the generation of the Embeddings vectors and finally the Graph Neural Network.

The neural network model used is the Graph Neural Network, as described in Section 7.2.3. Graph Neural Networks are trained for object recognition and classification in the scene, with the region graphs of images. For each of the region descriptions (shown in Table 4 for bees and in Table 5 for ants), a dataset is created; the graphs of each image in the dataset are converted into embedding vectors that serve as input to the neural network. The embedding vectors then contain all the features of the image and are converted into useful vector representations. The Graph Neural Network (GNN) computes the vector representations at each vertex of the input graph using a transition function *F*, which allows for computing the representation of the next neighborhood from the current representation; consider that the neighborhood size is variable, then apply the transition function is symmetrically. Figure 8 is a general representation of the training of the neural network for the recognition, classification, and description of objects in the scene.

The three components of the network are mentioned in the following list:

- Input to the neural network. The Graph Neural Network receives the complete scene graph of the image as input, generated from the bounding boxes; the object characteristics are obtained for the generation of vector embeddings as performed in [26].
- Processing. The Graph Neural Network receives the embedding vectors as input and carries out recognition of the objects in the scene.
- Neural network output. The output produced by the neural network is object recognition and classification.

The image dataset sizes for training the Graph Neural Network started with 5000 images for bees and 5000 images for ants, as it is considered a deep learning application. The maximum number of images used in training for each arthropod class was 25,000. These images are obtained from real-world settings over different time periods.

### 7.3. Evaluation of the Method

In [26], the method is evaluated in two ways: first, the system interacts with a user, and second, the system runs automatically. When the user interacts with the system, they choose an image and can freely formulate a question. The question is based on the regions of the image, or the set of questions proposed in Table 10 are used. Then, the scene graph is generated, and the resulting sentence or predicate is sent to the user. In contrast, when the system runs automatically, the host server receives the images remotely from the apiary, generates the scene graphs, and provides the results.

In this work, we performed the following experiments: first, automatic object recognition by the system for both types of arthropods, bees and ants; second, the generation of bounding boxes from the images and scene recognition with the generated sentences. Each experiment is described in Section 9 along with its results.

## 8. Architecture of Distributed Artificial Intelligence

Before describing the experiments that were carried out, this section presents the architecture of the distributed artificial intelligence designed and used in this research.

Numerous studies in the literature indicate that arthropods are susceptible to climate change. However, observing only a single nest provides limited information on the current status of a single colony. Therefore, this research proposes parallel, real-time observations of three geographically distributed sites. We begin with the following research question: is there a direct relationship between the behavior of arthropods (bees and ants) that inhabit colonies in geographically distributed sites with similar ambient temperatures and relative humidity?

This experiment proceeds as follows: A distribution of three prototypes (like the one shown in Figure 2) are divided into three different sites. At each of the three sites, each prototype acquires images of the nest entrances, which will allow us to understand the behavior of the different bee and ant colonies under certain climatic conditions, namely ambient temperature and relative humidity. We measure the impact on the behavior of each nest. For example, we observe whether the three nests behave the same when the ambient temperature is 14 degrees Celsius and the relative humidity is 42%.

To achieve this, the prototypes are installed at the observation sites, and then the images are acquired in the real environment and transmitted to the data processing center, and the massive behavior of the nests is determined using the descriptions presented in Table 4 and Table 5.

## 9. Experiments

As metrics are presented in reference [37] to determine the accuracy of image descriptions, in this work, we decided to start with evaluations of the techniques used for object recognition, bounding boxes, and scene recognition; in future works, other metrics will be used to measure the accuracy in graph reasoning for sentence detection, predicate classification, and scene graph classification [41]. Three parameters evaluated in this research work are the percentage of accuracy in the recognition of object, the percentage of accuracy in bounding boxes, and finally, the percentage of accuracy in scene recognition. The experiments are carried out with the “Bees” and “Ants” datasets separately. This evaluation allows us to determine the effectiveness of the method’s execution when it is executed in real time.

Fifteen datasets with different numbers of images were created for each evaluation; the first dataset contains 100 images, and the last one contains more than 1500 images. The same datasets were used for the evaluation of recognition, bounding boxes, and scene recognition.

### 9.1. Object Recognition

To evaluate the percentages of accuracy in the recognition of objects and their bounding boxes, the system receives the image and performs the detection of objects. In this experiment, $\left(S_{1}\right)$, the following must be true:

Statement 1 $\left(S_{1}\right)$, object recognition. Given *I*, obtain $o_{1},o_{2},\ldots ,o_{n}$, where $o_{1},o_{2},\ldots ,o_{n}\in \theta$; otherwise, any $o_{n}\notin \theta$.

The GNN recognizes the objects in the image. The detection threshold has been used for object recognition, and it is applied in the training of the neural network (as explained in Section 7.2.4); through the threshold, the recognition of the object in the scene is achieved, and it is classified as correct or incorrect.

**Figure 9:**
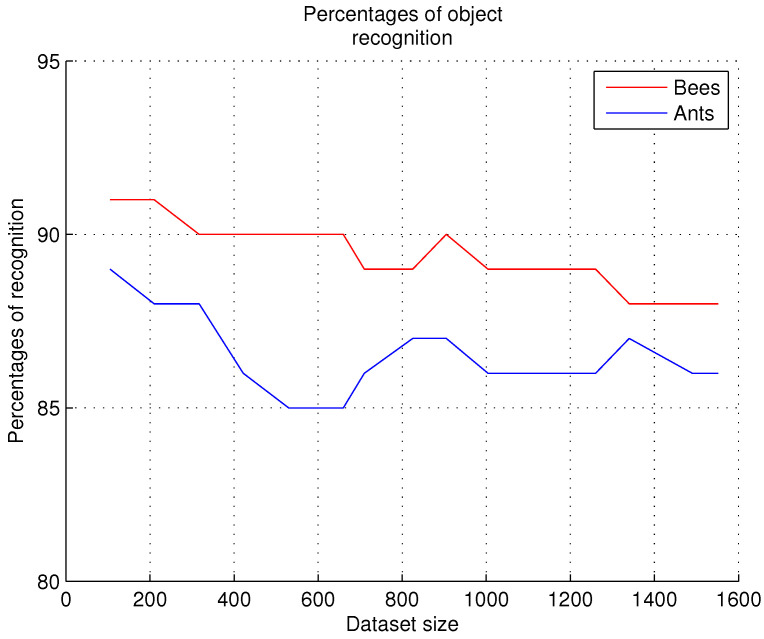
Percentages of accuracy in the recognition of objects and their bounding boxes.

For each execution of the system, with each of the datasets used, cases in which the program output is correct are counted, as well as cases in which the program does not output the correct result; the total for each of these cases is divided by the number of images in the dataset to obtain percentages of accuracy in the recognition of objects and their bounding boxes. Figure 9 shows the results obtained from the evaluations performed.

Comments. According to Figure 9, in these experiments, we observed that as the number of images in each dataset increases, the recognition rate remains stable in contiguous datasets and then decreases. To prevent a decrease in recognition accuracy, as shown in [26], we expanded the range of behaviors of bees and ants that the system needs to analyze. In other words, by incorporating more types of behavior, we were able to improve the accuracy of the recognition system. Additionally, to address the challenges posed by varying positions of bees and ants, the datasets were expanded with more images, and a more comprehensive catalog of images depicting intruders attempting to access the nests was included. However, special care must be taken when overtraining the neural network because increasing the number of training instances can cause the neural network to become blocked; the percentages reported in this research were obtained with images that represent most of the behaviors of arthropods exemplified in Table 3 and Table 4 of Section 7.2.2.

### 9.2. Bounding Boxes Evaluation

It is clear that the accuracy of bounding box detection leads to excellent generation of image predicates. Therefore, we consider it a high priority to conduct the experiment to evaluate the accuracy of the bounding boxes generated in the images. For the above, Statement 2 is evaluated as follows:

Statement 2 $\left(S_{2}\right)$, object relationships. If $S_{1}$ is true, then for each $o_{1},o_{2},\ldots ,o_{n}$, create a graph *G* numbered $G_{1},G_{2},\ldots ,G_{n}$ and create the bounding boxes contained in the image. Find a relationship $R_{1},R_{2},\ldots ,R_{n}$ between objects.

The bounding boxes evaluated correspond to the 10 frames that can be generated for bees and are shown in Table 6. For ants, the creation of the five bounding boxes shown in Table 7 was evaluated.

**Figure 10:**
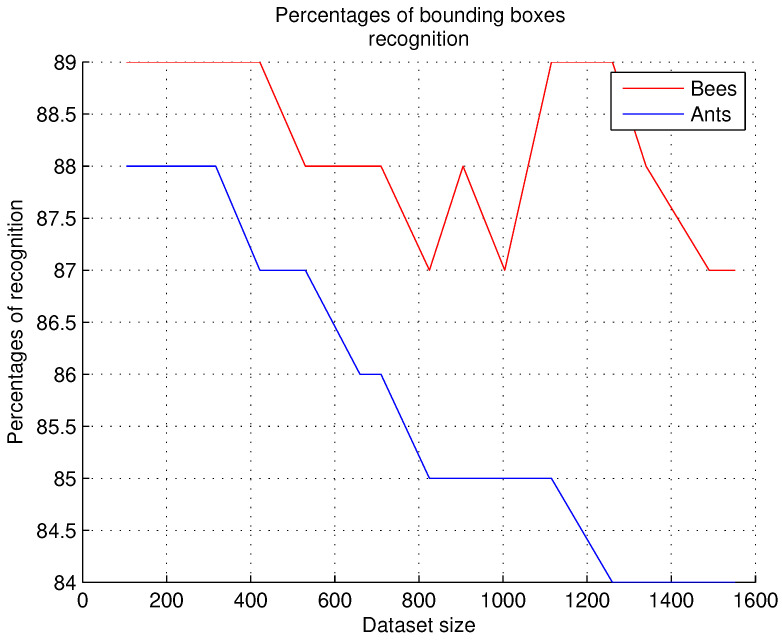
Evaluation of bounding boxes.

This experiment is performed as follows: an image dataset containing the bounding boxes is trained; then, the system receives as input the images of the observation sites (established in the 15 datasets represented in the Figure 10 on the *X* axis), detects the objects in the image, produces the graph, and generates the bounding boxes in the received image.

To evaluate the accuracy of the result, that is, the detection of the bounding boxes in the image, it is verified against the training image dataset; this verification procedure allows us to determine the accuracy of detecting at least four out of six bounding boxes in the image. If four or five bounding boxes are detected, the experiment is considered correct; if only three or fewer bounding boxes are detected, the detection process is considered failed.

We defined the success accuracy (threshold = 4) as a probability greater than 0.5. That is, if there are six bounding boxes, considering only three of them only achieves a probability of 0.5, which means a 50% success rate and a 50% failure rate. This is also supported by the experiments performed: when only three bounding boxes were considered, the number of false positives increased. Figure 10 shows the recognition percentages obtained with 15 datasets.

Comments. According to Figure 10, recognition rates of up to 89% were obtained in these experiments when the bees’ bounding boxes were obtained. In the case of ants, the recognition rates for bounding boxes are lower; the highest rates reach a maximum of 88%. We observed that only images showing new positions of bees or ants are not recognized by the neural network; for example, when bees fly over the entrance to the nest, and in the case of ants, bounding boxes are not generated when the objects overlap. When new positions of these arthropods are found, they are then included in the training dataset for future recognition.

### 9.3. Scene Recognition

Once the two previous experiments have been carried out, we are able to perform scene recognition to obtain a textual description of the image. Then, statement 3 is evaluated:

Statement 3 $\left(S_{3}\right)$, textual description. If and only if $S_{1}$ and $S_{2}$ are true, then the process of building a scene graph is realized and a formal description of *I* is generated.

In these experiments, we evaluated the accuracy percentages in scene recognition. The results were obtained as follows: when the system categorizes the predicate for each object pair, it is determined whether the predicate is correct for the image or incorrect, using Visual Context Input for SGG, as described in Section 5.2.

**Figure 11:**
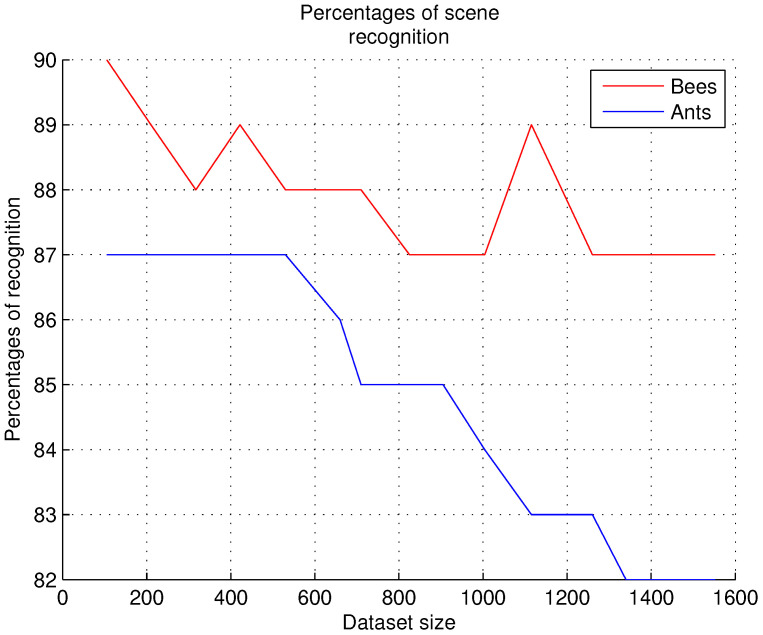
Evaluation of scene recognition.

To determine whether a predicate is correct, we use Training Loss, as defined in Section 5.2. For training loss, we used the default threshold as a binary classification; the standard threshold is 0.5. If the predicted probability is greater than 0.5, the instance is classified as positive (1); otherwise, it is classified as negative (0). The predicates evaluated as correct and the predicates evaluated as incorrect are counted for each dataset evaluated; the number of correct predicate evaluations is divided by the number of images recognized in the dataset to obtain the percentages of accuracy in the scene. Figure 11 shows the scene recognition percentages obtained with 15 datasets.

In this experiment, predicate categorization rates for each object pair were identified to be as high as 90% for the bees and as high as 87% for the ants, as shown in Figure 11. To ensure 90% recognition, it was necessary to achieve similar percentages in the two previous experiments. We can conclude from this experiment that the 10% non-recognition rate for the bees is due to complex scenes that show overlapping colony individuals or because the nest entrance remains empty. Other factors, such as a cloudy day or shadows cast by the prototype at the nest entrance, are also causes of scene non-recognition. This experiment also allowed us to identify how the triplet 〈subject−predicate−object〉 (we refer to Section 7.2.3) is constructed and to determine whether this triplet is appropriate for the scenes evaluated in each of the images.

## 10. Conclusions

In this research, we conducted an investigation aiming to observe the behavior and organization of two types of insects, bees and ants, using nest access points as observation areas. A hardware system was developed to obtain visual and environmental information from the research sites, namely, anthills and apiaries. The hardware system was complemented by a software system that uses artificial intelligence techniques, specifically scene graphs, to recognize objects in the scene, determine relationships between objects, and construct a textual description per image. Through this research, we demonstrate that current technology allows us to preserve and care for endangered species while also preventing the mass killing of insects currently carried out for the purpose of analyzing and studying endemic species. In an extensive literature review, we found no computational system for recognizing behavioral and organizational aspects of bees and ants using the techniques proposed in this research. Extensive experiments were conducted in real-life environments, including access to anthills and beehives. The results of the experiments demonstrate the feasibility of the proposed software system and hardware architecture for recognizing, classifying, and generating textual descriptions of the images obtained.

## 11. Discussion

In this research, we limited our study to object recognition techniques, bounding boxes, and scene recognition, applied to real arthropod environments, and we measured their accuracy through a series of experiments. Currently, the lead author of this work is conducting research on the application of mathematical models that will allow for measuring the accuracy of graph reasoning for sentence detection, predicate classification, and scene graphical classification. These mathematical models propose the use of environmental variables such as temperature and humidity, which modify the behavior of arthropods.

This work is part of a series of published manuscripts in which a real-time system (observation prototype) continuously monitors and collects data on the life cycles of bees and ants to extract behavioral characteristics. Through this research, we aim to understand and anticipate the risks of extinction or disease that these species face.

As a final note, referring to the work of [47] titled “Predictability: Does the Flap of a Butterfly’s Wings in Brazil Set Off a Tornado in Texas?”, we note that if a butterfly is eliminated by conducting studies that capture hundreds of insects to be killed and then observed, the tornado will surely not reach Texas. In the century in which we exist, it is no longer justifiable to eliminate insects to conduct a study; current technologies offer us the opportunity to automate processes, to visualize what our eyes cannot see, but we must develop the systems to do so.

## 12. Patents

A patent for the observation prototype is in process.

## Figures and Tables

**Table 1:** Geographical locations and aerial photos of four anthill sampling sites. Anthill 1, is an area between a corn crop and a bean crop area. Anthill 2 is near a tree and next to pig and sheep farms. Anthill 3, is located in an area with cactus plants, without access to the crops. Anthill 4, near a building and walkways for people.

Image 1. Anthill 1	Image 2. Anthill 2
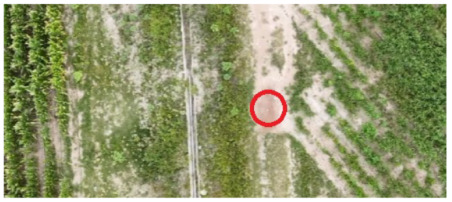	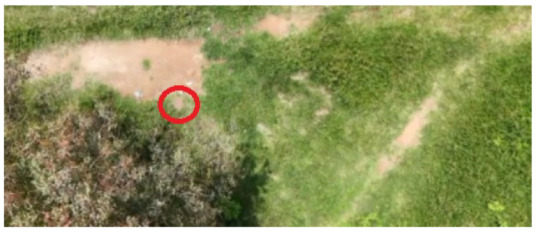
Image 3. Anthill 3	Image 4. Anthill 4
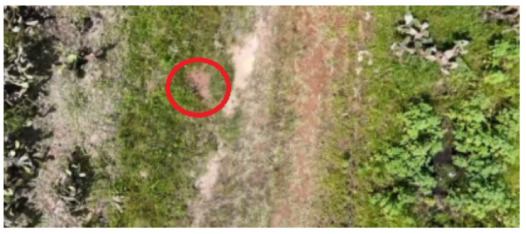	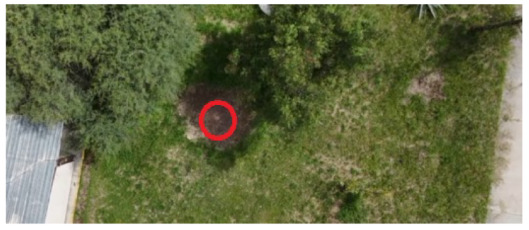

**Table 2:** Examples of images acquired from the test apiaries. Each image in the table is identified with a number and a name related to the behavior shown by the bees.

Image 1. Greeting: two bees greeting	Image 2. Type of bee
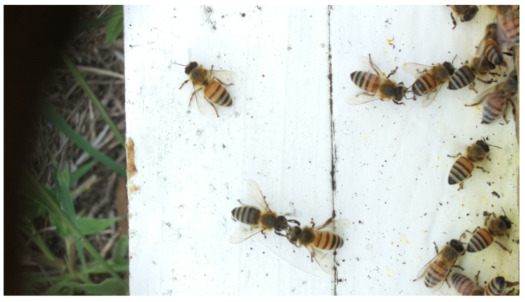	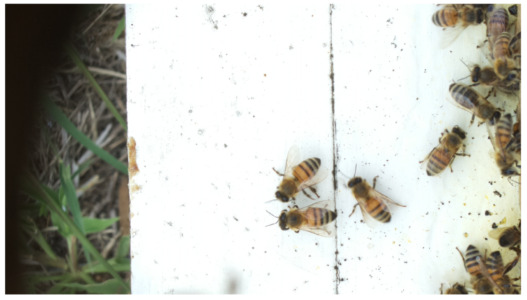
Image 3. Carrying food	Image 4. Intruder
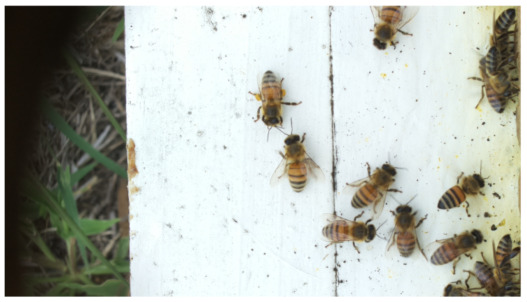	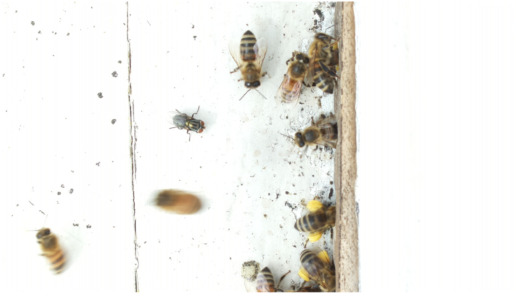
Image 5. Big population	Image 6. Grouping
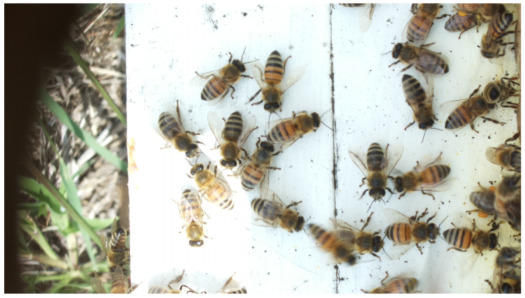	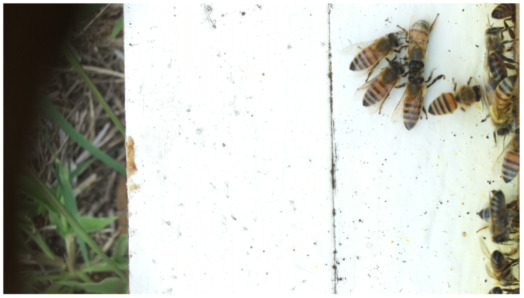

**Table 3:** Examples of images acquired from the anthills. Each image in the table is identified with a number and a name related to the behavior shown by the ants.

Image 1. Greeting	Image 2. Type of ant
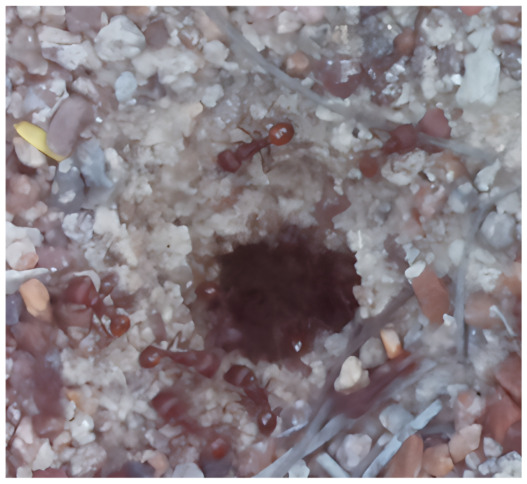	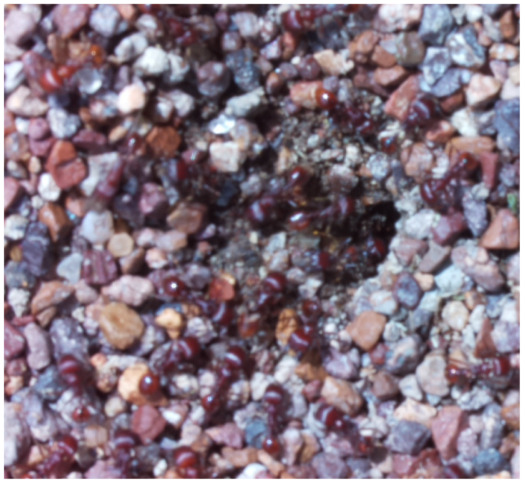
Image 3. Carrying food	Image 4. Intruder
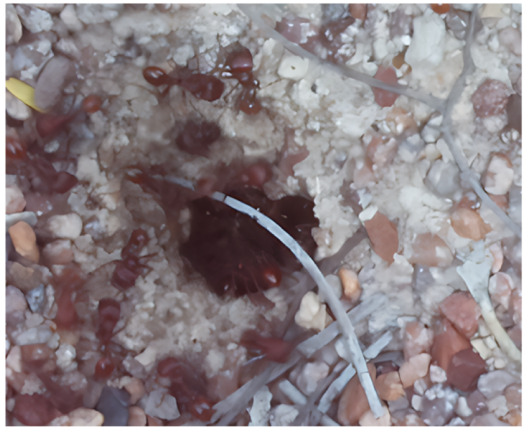	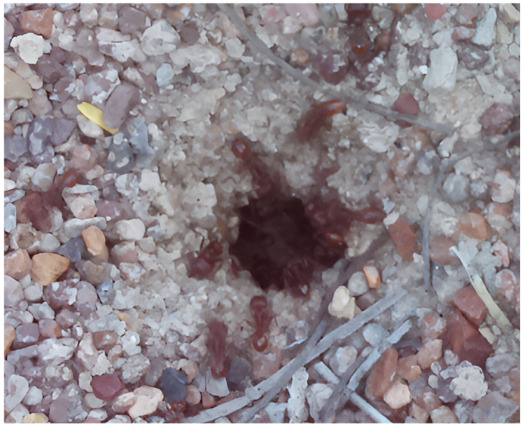
Image 5. Big population	Image 6. Grouping
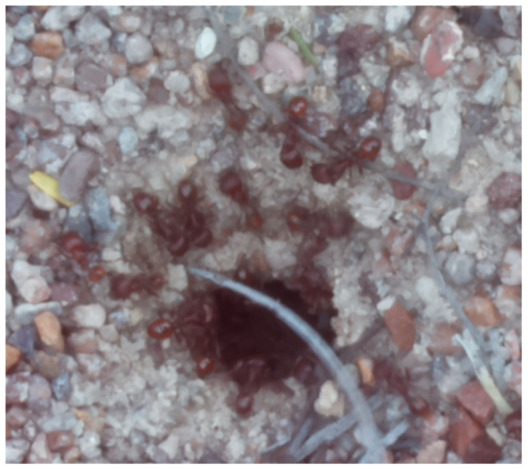	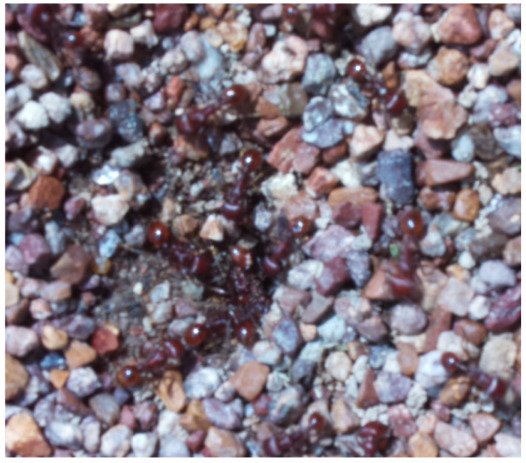

**Table 4:** Descriptions of the regions of two scenes from the Table 2: Greeting and Intruder.

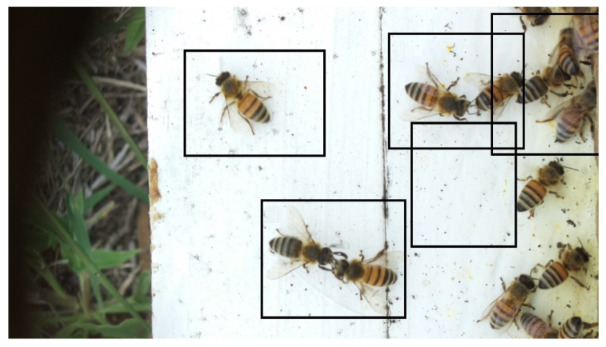	Bee Greeting Beehive Entrance Bee Take-Off Platform Isolated Bee at Beehive Entrance Bees Loitering
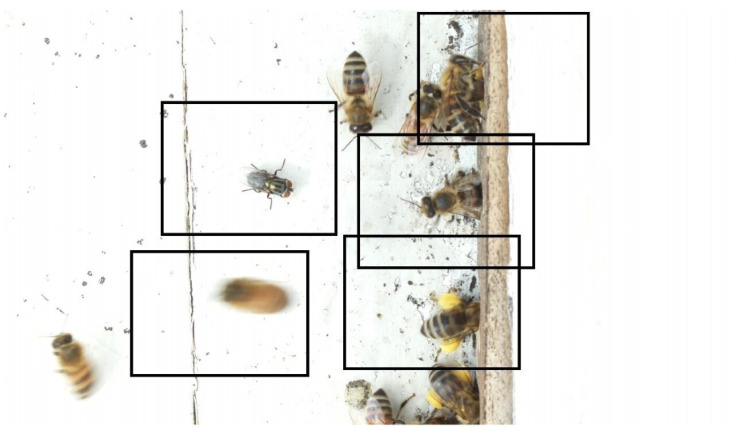	Intruder Detected Beehive Exit Carrying Pollen Bee Flying Bee in Lateral Position

**Table 5:** Descriptions of the regions of two scenes from the Table 3: Greeting and Big Population.

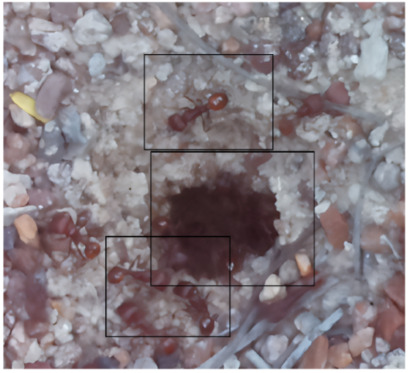	Ant Greeting Anthill Entrance Isolated Ant at Anthill
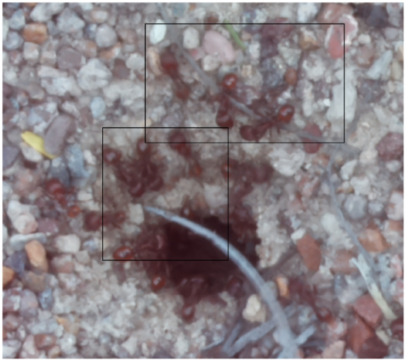	Carrying Food Ants Loitering

**Table 6:** Mapping of the phrases to the bounding boxes of the two images in Table 1.

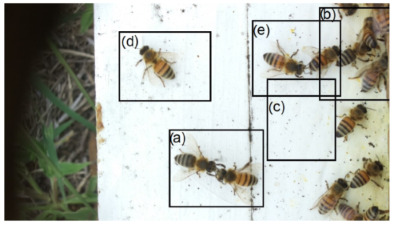	(a) Bee greeting (b) Beehive access (c) Bee take-off platform (d) Isolated bee at the entrance (e) Bees loitering
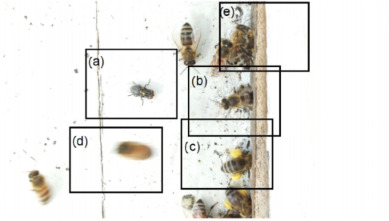	(a) Intruder detected (b) Beehive exit (c) Carrying pollen (d) Bee flying (e) Bee in lateral position

**Table 7:** In both images, the bounding boxes are mapped to the phrases of two images of ants in Table 3. In the first image, the labels (a), (b), and (c) of the bounding boxes are used to map the three phrases with the same labels. In the second image, two labels are used.

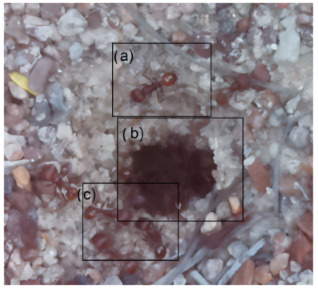	(a) Isolated ant at the entrance (b) Anthill access (c) Ant greeting
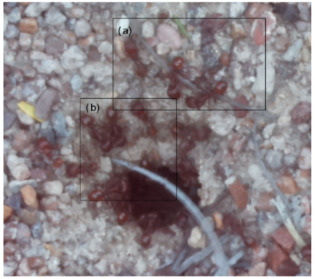	(a) Carrying food (b) Ants loitering

**Table 8:** Objects and attributes associated with objects (bees).

Object	Attributes Associated with Objects		
	Color	State	Position
Bee	Yellow	Flying	Raised wings
	Black	Accessing	Wings at rest
	Grey	Leaving	Next to
Intruder	Grey	Vigilant	Raised wings
Access	Free	Without bees	Vertical
	Occupied	Low population	Vertical
	Tumult	High population	Vertical

**Table 9:** Objects and attributes associated with objects (ants).

Object	Attributes Associated with Objects		
	Color	State	Position
Ant	Red	Walking	Raising antennas
	Yellow	Accessing	Detained and guarded
	Black	Leaving	Next to
Intruder	Grey	Vigilant	Raised wings
Ant	Free	Without ants	Vertical
	Occupied	Low population	Vertical
	Tumult	High population	Vertical

**Table 10:** Set of free-form questions and answers based on the complete image.

Question	Answer
How is the access to the beehive?	Big population
What are the bees doing at the entrance to the hive?	Grouping
Who is at the entrance of the hive?	Intruder
Why are the bees working?	Carring pollen
What is in the beehive entrance?	Type of bee

## Data Availability

Data available upon request due to privacy restrictions. The data presented in this study are available upon request to the corresponding author. The data are not publicly available because the images are acquired in real time from the entrances to the bee and ant nests and are not processed with any software, meaning that the size of each image is too large for transmission to various media. However, they are available to interested authors, provided that the authorship of this work is cited.
